# Nitazoxanide Modulates Mitochondrial Function and Inflammatory Metabolism in Chondrocytes from Patients with Osteoarthritis via AMPK/mTORC1 Signaling

**DOI:** 10.3390/antiox14050512

**Published:** 2025-04-24

**Authors:** Ha Eun Kim, Jong Yeong Lee, Ga-Yeon Son, Jun-Young Park, Ki Bum Kim, Chul-Min Choi, Young Jae Moon, Jin Kyeong Choi

**Affiliations:** 1 Department of Immunology, Jeonbuk National University Medical School, Jeonju 54907, Republic of Korea; gkdms21106@gmail.com (H.E.K.); shutp100@hanmail.net (J.Y.L.); 2 Department of Molecular Pathobiology, New York University College of Dentistry, New York, NY 10010, USA; gys1@nyu.edu; 3 Department of Biochemistry, Chungbuk National University, Cheongju 28644, Republic of Korea; jypark919@cbnu.ac.kr; 4 Department of Orthopedic Surgery, Jeonbuk National University Medical School and Hospital, Jeonju 54896, Republic of Korea; tibikim@naver.com; 5 Department of Healthcare, Dongguk University Duica, Seoul 04620, Republic of Korea; bradchoi@dongguk.edu; 6 Department of Biochemistry and Molecular Biology, Jeonbuk National University Medical School, Jeonju 54896, Republic of Korea; 7 Biomedical Research Institute, Jeonbuk National University Hospital, Institute for Medical Sciences, Jeonbuk National University, Jeonju 54907, Republic of Korea

**Keywords:** nitazoxanide, osteoarthritis, chondrocytes, AMPK, mTORC1, mitochondrial dysfunction, mitochondrial reactive oxygen species

## Abstract

Osteoarthritis (OA) is a long-term degenerative condition of the joints, characterized by persistent inflammation, progressive cartilage breakdown, and impaired mitochondrial function. Recent studies have shown that hyperactivation of the mTORC1 pathway and metabolic reprogramming of chondrocytes contribute to disease progression. Nitazoxanide (NTZ), an oral antiparasitic agent approved by the Food and Drug Administration, has shown anti-inflammatory and mitochondrial protective effects in various disease situations; despite this, its application in osteoarthritis has yet to be fully investigated. Here, we assessed the therapeutic efficacy of NTZ using IL-1β-stimulated primary chondrocytes derived from patients with OA. NTZ substantially reduced the expression of proinflammatory cytokines and matrix metalloproteinases, restored mitochondrial membrane potential, and reduced mitochondrial reactive oxygen species levels. NTZ also effectively reversed IL-1β-induced glycolytic metabolic changes by inhibiting glucose uptake and GLUT1 expression. Mechanistically, NTZ inhibited the activation of the mTORC1 pathway and substantially increased AMPK phosphorylation. The siRNA-mediated AMPK knockdown negated NTZ-induced mitochondrial and metabolic improvements, suggesting that AMPK is a key upstream regulator of the protective actions of NTZ. NTZ can, therefore, effectively inhibit inflammatory metabolic reprogramming and mitochondrial dysfunction in OA chondrocytes through AMPK-dependent mTORC1 signaling inhibition, highlighting its potential as a disease-modifying therapy for OA.

## 1. Introduction

Osteoarthritis (OA) is the most common degenerative joint disease causing joint pain, stiffness, and loss of function; its prevalence is steadily increasing worldwide [1]. Although OA was initially considered a disease limited to cartilage breakdown, recent studies have suggested that it is a systemic inflammatory disease that involves a combination of structural changes and chronic inflammation throughout the joint [2]. OA is a complex pathology that affects multiple joint components, including cartilage, subchondral bone, synovium, and the infrapatellar fat pad, as well as meniscal degeneration, which is not only fibrotic but also inflamed [3,4,5].

Early onset of OA is associated with various etiologies, including aging, metabolic disease, and trauma, which lead to cartilage degradation through proteoglycan and collagen loss, chondrocyte stress, and altered subchondral bone remodeling [2]. In particular, matrix metalloproteinases (MMPs) and inflammatory mediators, originating from both chondrocytes and synovial membrane cells, further deteriorate chondrocyte function through autocrine or peripheral cell-secretory mechanisms and act on the adjacent synovium to induce proliferation and inflammatory responses [6].

Inflammatory cytokines (such as tumor necrosis factor (TNF)-α, interleukin (IL)-1β, and IL-6) are produced not only by chondrocytes but also by the synovial membrane and infrapatellar fat pad under pathological conditions [7]. Impaired mitochondrial function induces the production of reactive oxygen species (ROS), which reactivates the inflammatory response within chondrocytes, accelerating the onset and progression of OA [7]. This inflammatory response exacerbates the mitochondrial pathology, creating a vicious cycle of chondrocyte dysfunction and joint degeneration [7].

Recently, the mammalian target of rapamycin (mTOR) pathway has been recognized as an important metabolic signaling axis in OA pathophysiology [8]. One of its subcomplexes, mTOR complex 1 (mTORC1), is a major signaling pathway that governs cellular proliferation, protein production, and maintenance of energy metabolic balance and is tightly regulated in normal chondrocytes in response to nutrient status and energy balance [9]. However, in the setting of OA, overactivation of the mTORC1 pathway has been reported to promote chondrocyte degradation, amplify inflammatory responses, cause mitochondrial damage, and accelerate cellular senescence [8,9]. Thus, inhibition of mTORC1 may be a promising therapeutic strategy for modulating OA progression.

Current treatments for OA focus on symptomatic relief of pain and inflammation and include NSAIDs, intra-articular corticosteroids, and structured physical therapy [8,10]. However, these treatments only provide temporary symptomatic relief and do not inhibit underlying disease progression; severe cases require total joint replacement, which has limitations in terms of cost and prognosis [2]. Therefore, elucidating the pathogenesis of OA and developing new therapies that can effectively inhibit its progression is imperative.

Nitazoxanide (NTZ) is a Food and Drug Administration (FDA)-approved oral antiparasitic drug of the thiazolide class, whose biological functions, such as inhibition of inflammation, reduction of ROS, inhibition of mTOR, induction of autophagy, and regulation of energy metabolism, have recently been reported in various studies including Parkinson’s disease, *Taenia crassiceps* infection, rheumatoid arthritis fibroblast-like synoviocytes, psoriasis, and atherosclerosis [10,11,12,13,14,15]. In rheumatoid arthritis models, NTZ inhibits the proliferation and inflammatory response of synoviocytes [16]. In the present study, we evaluated whether NTZ could inhibit IL-1β-induced inflammatory metabolic reprogramming and mitochondrial dysfunction in primary chondrocytes derived from patients with OA. We aimed to demonstrate that NTZ could alleviate the pathological conditions in OA chondrocytes by inducing mitochondrial function recovery, oxidative stress reduction, and metabolic balance regulation through AMPK activation and mTORC1 inhibition.

## 2. Materials and Methods

### 2.1. Chemicals

Nitazoxanide (NTZ; Cat# S1629) was obtained from Sigma-Aldrich (St. Louis, MO, USA), and rapamycin (Cat# R-5000) was purchased from LC Laboratories (Woburn, MA, USA). The chemical structure of NTZ is presented in Supplementary Figure S1a.

### 2.2. Chondrocyte Isolation from Patients

Primary chondrocytes were obtained from osteoarthritic cartilage collected during total knee replacement surgery in three independent OA patients, aged 63–80 years, at Jeonbuk National University Hospital. All experiments were performed using biological replicates derived from these individual donors. This study was approved by the Institutional Review Board of the Jeonbuk National University Hospital (IRB No. 2021-02-010-005). Cartilage samples were minced in serum-free Dulbecco’s modified Eagle medium (SF DMEM; Welgene, Kyungsan-si, Republic of Korea) containing hyaluronidase (Cat# H3506, Sigma-Aldrich, St. Louis, MO, USA). The minced cartilage tissues were first incubated in serum-free DMEM containing protease from *Streptomyces griseus* (Type XIV, ≥3.5 units/mg solid, Sigma-Aldrich, St. Louis, MO, USA, Cat# P5147) at a concentration of 5 mg/mL at 37 °C for 1 h. This was followed by a second digestion step in serum-free DMEM containing hyaluronidase (Cat# H3506, Sigma-Aldrich, St. Louis, MO, USA, 600 U/mL) and collagenase from *Clostridium histolyticum* (Type II, ≥125–250 units/mg, Sigma-Aldrich, St. Louis, MO, USA, Cat# C9263) at a concentration of 2 mg/mL at 37 °C for 3 h. The digested tissue suspension was filtered through a 70 μm cell strainer to isolate single chondrocytes, which were then cultured in DMEM supplemented with 20% fetal bovine serum (FBS; Gibco, Grand Island, NY, USA) and antibiotics (100 U/mL penicillin G and 100 μg/mL streptomycin) at 37 °C in a humidified incubator with 5% CO_2_.

### 2.3. Cell Culture and Stimulation

To stimulate chondrocytes, cells were treated with 10 ng/mL recombinant IL-1β (Cat# 200-01B, PeproTech, Rocky Hill, NJ, USA) either alone or co-treated simultaneously with 25 or 50 μM NTZ for 24 h. In parallel experiments, rapamycin (1 μM) was co-administered with IL-1β under the same conditions.

### 2.4. Cell Viability Assay

Cell viability was assessed using the MTT assay, which measures mitochondrial metabolic activity. Chondrocytes were exposed to varying concentrations of NTZ (0 to 1000 μM) or rapamycin (0.1, 1, and 10 μM) for 24 h. Following treatment, MTT reagent was added directly to the culture medium, and the cells were incubated at 37 °C for 3 h to allow for formazan crystal formation. The resulting crystals were dissolved in dimethyl sulfoxide (DMSO), and absorbance was recorded at 570 nm using a microplate reader.

### 2.5. Quantitative Real-Time PCR (qPCR)

Total RNA was isolated from cultured chondrocytes using RNAiso Plus reagent (Cat# 9109, Takara, Tokyo, Shiga, Japan), following the manufacturer’s protocol. After phenol/chloroform purification, RNA was dissolved in RNase-free water (Thermo Fisher Scientific, Waltham, MA, USA). First-strand complementary DNA (cDNA) was synthesized using the RevertAid First Strand cDNA Synthesis Kit (Cat# 1622, Thermo Fisher Scientific, Waltham, MA, USA). Quantitative real-time PCR (qPCR) was carried out using the QuantStudio™ 5 Real-Time PCR Instrument (Applied Biosystems, Thermo Fisher Scientific, Waltham, MA, USA). Each reaction mixture contained SYBR Green PCR Master Mix (NANOHELIX, Daejeon, Republic of Korea) and gene-specific primers listed in Table S1. PCR conditions followed the manufacturer’s recommendations. Expression levels of target genes were normalized to β-actin, and relative quantification was performed using the 2^−ΔΔCt^ method.

### 2.6. Western Blotting

Cell lysates were prepared using RIPA buffer (Thermo Fisher Scientific, Waltham, MA, USA, Cat# 89900) supplemented with a protease inhibitor cocktail (cOmplete™, Mini, EDTA-free, Roche, Basel, Switzerland Cat# 11836170001) and a phosphatase inhibitor cocktail (PhosSTOP™, Sigma-Aldrich, distributed by Roche, Cat# 04906845001). Proteins were separated by molecular weight using sodium dodecyl sulfate–polyacrylamide gel electrophoresis (SDS-PAGE; 6%, 8%, 10%, and 12%) and transferred to nitrocellulose membranes. Western blotting was performed as previously described [17]. All primary antibodies were prepared in 1×TBST containing 5% BSA (Fraction V; Sigma-Aldrich, St. Louis, MO, USA, Cat# A7906) and applied at optimized dilutions. Specifically, phospho-mTOR (clone D9C2) and total mTOR (clone 7C10), both from Cell Signaling Technology (CST, Danvers, MA, USA), were used at 1:1000. Antibodies against MMP1 (1:1500), MMP3 (1:1000), MMP9 (1:2000), and MMP13 (1:1500) (Thermo Fisher Scientific) were used at their respective dilutions. HRP-conjugated secondary antibody targeting rabbit IgG (ADI-SAB-300, Enzo Life Sciences, Farmingdale, NY, USA) was used at a dilution of 1:10,000. Signal detection was performed using the Amersham Imager 600 (Cytiva, Marlborough, MA, USA) in combination with an enhanced chemiluminescence (ECL) substrate. Band intensities were quantified by densitometric analysis using ImageJ software (version 1.53k, NIH, Bethesda, MD, USA). The intensity of each phospho-protein band was normalized to its corresponding total protein band, and MMP levels were normalized to β-actin.

### 2.7. Oxygen Consumption Rate (OCR)

Chondrocytes were seeded at a density of 2 × 10^4^ cells per well onto poly-D-lysine–coated Seahorse XFp miniplates (Agilent Technologies, Santa Clara, CA, USA) and preincubated in XF assay medium supplemented with 1.5 μM oligomycin, 0.5 μM FCCP, and 0.5 μM rotenone/antimycin A. To evaluate mitochondrial respiration, the oxygen consumption rate (OCR) was measured using the XFp Cell Mito Stress Test Kit in accordance with the manufacturer’s protocol. Data were acquired with the Seahorse XFp Analyzer (Agilent Technologies).

### 2.8. Extracellular Acidification Rate (ECAR)

Chondrocytes were seeded at a density of 2 × 10^4^ cells per well in a Seahorse-compatible medium supplemented with 10 mM glucose for extracellular acidification rate (ECAR) analysis. Glycolytic function was evaluated using the XF Glycolytic Stress Test Kit (Agilent Technologies, Santa Clara, CA, USA), following the manufacturer’s protocol. ECAR was recorded with a Seahorse XFp Analyzer (Agilent Technologies).

### 2.9. Glucose Uptake Assay

Chondrocytes were plated at a density of 5 × 10^4^ cells per well in 96-well flat-bottom plates, using DMEM as the culture medium. To evaluate glucose uptake, cells were treated with 2-NBDG (0.01 mg/mL; Cat# N13195, Thermo Fisher Scientific, Waltham, MA, USA) and incubated for 30 min at 37 °C. Following treatment, cells were washed twice with phosphate-buffered saline (PBS) and subsequently labeled with Live/Dead Fixable Viability Dye (Cat# L34962, Thermo Fisher Scientific, Waltham, MA, USA) for 20 min at room temperature. Fluorescence signals were acquired and analyzed using the Attune NxT flow cytometer (Thermo Fisher Scientific, Waltham, MA, USA).

### 2.10. Mitochondrial Content Measurement

Chondrocytes (5 × 10^4^ cells/well) were seeded in DMEM in 96-well flat-bottomed plates. Following treatment with 0.25% trypsin, the cells were transferred to 96-well U-bottom plates and incubated with either MitoTracker Green FM (Cat# M7514) or MitoSOX (Cat# M36008) Red (both from Thermo Fisher Scientific, Waltham, MA, USA) at 37 °C for 1 h. After incubation, the cells were washed twice with 1× PBS and stained with Live/Dead Fixable Dead Cell Dye (Thermo Fisher Scientific, Waltham, MA, USA) for 20 min at room temperature to detect viable cells. The samples were analyzed using an Attune NxT acoustic focus cytometer (Thermo Fisher Scientific Waltham, MA, USA).

### 2.11. siRNA Transfection

To silence AMPK expression, human primary chondrocytes were transfected with siRNA targeting AMPKα1/2 (Cat# sc-45312, Santa Cruz Biotechnology, Dallas, TX, USA) or a non-targeting control siRNA (Cat# sc-37007) using the FuGENE^®^ HD transfection reagent (Promega, Madison, WI, USA). The lyophilized siRNA was reconstituted in siRNA dilution buffer (Santa Cruz Biotechnology) to a final concentration of 10 µM. For transfection complex formation, 101 µL of transfection medium was combined with 24 µL of FuGENE reagent (Solution A), and 8 µL of siRNA (10 µM stock) was diluted in 117 µL of transfection medium (Solution B). Solution A was then added dropwise to Solution B, gently mixed, and incubated at room temperature for 15 min to allow complex formation (Solution C). This siRNA-FuGENE complex was added evenly to cells cultured in 6-well plates, followed by gentle rocking to ensure uniform distribution. The transfected cells were incubated at 37 °C with 5% CO_2_ for 83 h, after which IL-1β (10 ng/mL) and NTZ (25 or 50 μM) treatments were applied as indicated for subsequent analyses.

### 2.12. Immunofluorescence Imaging

Chondrocytes were seeded at a density of 1 × 10^5^ cells/well onto Millicell EZ glass slides (Cat# PEZGS0896, Sigma-Aldrich, St. Louis, MO, USA) and cultured overnight. The next day, cells were exposed to IL-1β, nitazoxanide (NTZ), or rapamycin for 24 h. Following treatment, the culture medium was replaced with DMEM containing MitoTracker Green FM and MitoSOX Red (both from Thermo Fisher Scientific), and the cells were incubated at 37 °C for 1 h. After incubation, cells were rinsed with Dulbecco’s phosphate-buffered saline (DPBS) and fixed in 4% paraformaldehyde at 4 °C for 15 min. Subsequently, cells were permeabilized using 0.3% Triton X-100 for 10 min at room temperature in the dark. After washing, nuclear staining was performed with DAPI solution for 5 min, followed by DPBS rinsing. Coverslips were mounted using a fluorescent mounting medium (Cat# S302380-2, Agilent Technologies, Santa Clara, CA, USA). Fluorescence images were captured at 200× magnification using the ZEN imaging system (ZEISS, Jena, Germany). The detection ranges used for each fluorophore were: DAPI: 410–577 nm; MitoSOX Red: 576–753 nm; MitoTracker Green: 490–582 nm.

### 2.13. Statistical Analysis

Statistical analyses were carried out using GraphPad Prism version 9.5.0 (GraphPad Software, San Diego, CA, USA). Depending on the experimental design, either an unpaired Student’s *t*-test or one-way ANOVA followed by Holm–Šídák’s post hoc test was employed to compare group differences. Data are presented as mean ± standard error of the mean (SEM), and significance levels were defined as: * *p* < 0.05; ** *p* < 0.01; *** *p* < 0.001; **** *p* < 0.0001.

## 3. Results

### 3.1. NTZ Attenuates IL-1β-Induced Inflammatory Responses and MMP Expression in OA Chondrocytes

**Figure 1:**
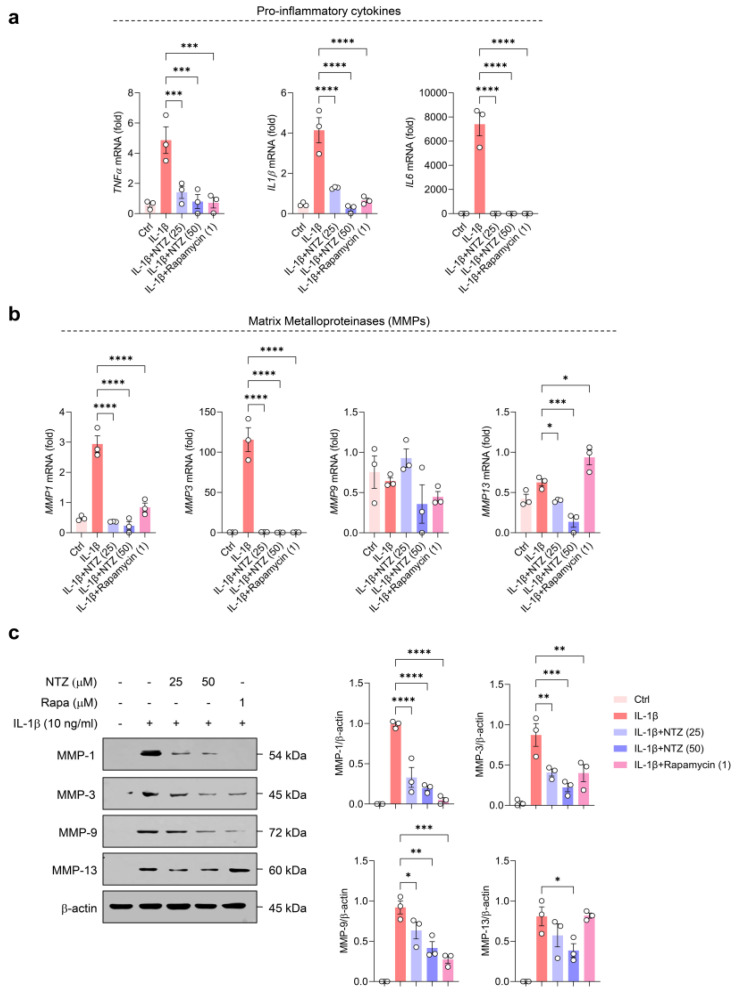
Nitazoxanide (NTZ) suppresses interleukin (IL)-1β-induced proinflammatory cytokines and matrix metalloproteinase (MMP) expression in osteoarthritis (OA) chondrocytes. Primary human chondrocytes derived from osteoarthritis (OA) patients were exposed to IL-1β (10 ng/mL) for 24 h, with or without co-treatment with nitazoxanide (NTZ; 25 or 50 μM) or rapamycin (1 μM). (**a**) Transcript levels of pro-inflammatory cytokines, including tumor necrosis factor-α (*TNF*-*α*), *IL*-*1β*, and *IL*-*6*, were measured using quantitative real-time PCR (qPCR). (**b**) The gene expression of matrix-degrading enzymes (*MMP1*, *MMP3*, *MMP9*, and *MMP13*) was also evaluated by qPCR. (**c**) Protein expression of MMP1, MMP3, MMP9, and MMP13 was analyzed via western blot. Band intensities were quantified through densitometric analysis using ImageJ software and are shown as bar graphs. Statistical comparisons were performed using Holm-Šídák’s post hoc test. Data are presented as mean ± standard error of the mean (SEM). * *p* < 0.05, ** *p* < 0.01, *** *p* < 0.001, and **** *p* < 0.0001 compared to the IL-1β-stimulated group.

To examine the non-cytotoxic concentration ranges of NTZ and rapamycin in primary OA chondrocytes, MTT assays were performed after treatment with various concentrations of each drug for 24 h. Concentrations of 25 and 50 µM of NTZ and 1 µM of rapamycin were used for subsequent experiments, as these concentrations maintained cell viability above 80% (Figure S1b). IL-1β is well known as a major inflammatory cytokine that induces cartilage degradation and catabolic reactions in OA chondrocytes [18]. To evaluate the anti-inflammatory and matrix-protective effects of NTZ, primary chondrocytes derived from patients with OA were stimulated with IL-1β (10 ng/mL) and treated with NTZ (25 and 50 µM) or rapamycin (1 µM). The qPCR analysis showed that IL-1β substantially increased the gene expression levels of TNF-α, IL-1β, and IL-6, and NTZ effectively inhibited the expression of these cytokines to a level similar to that of rapamycin (Figure 1a). Furthermore, analysis of the expression of MMPs involved in cartilage matrix degradation showed that IL-1β stimulation substantially increased the mRNA expression of MMP1, MMP3, MMP9, and MMP13, whereas NTZ substantially inhibited the expression of MMP1, MMP3, and MMP13 but not MMP9 mRNA (Figure 1b). Western blot analysis showed that the protein expression levels of MMP1, MMP3, MMP9, and MMP13 were consistently decreased by NTZ treatment, suggesting the possibility of post-transcriptional regulation (Figure 1c). Although MMP9 mRNA expression showed modest changes upon IL-1β stimulation, Western blot analysis revealed a clear upregulation at the protein level, suggesting potential post-transcriptional regulation. Densitometric analysis indicated a marked decrease in the expression of all MMP proteins, and the inhibitory potency was equivalent to or, in some cases, superior to that of rapamycin. These results demonstrate that NTZ effectively inhibited IL-1β-induced inflammatory responses and MMP expression in OA chondrocytes, suggesting that NTZ may be a promising disease-modifying agent for the treatment of OA.

### 3.2. NTZ Inhibits IL-1β-Induced Glycolysis-Biased Metabolic Changes in OA Chondrocytes

**Figure 2:**
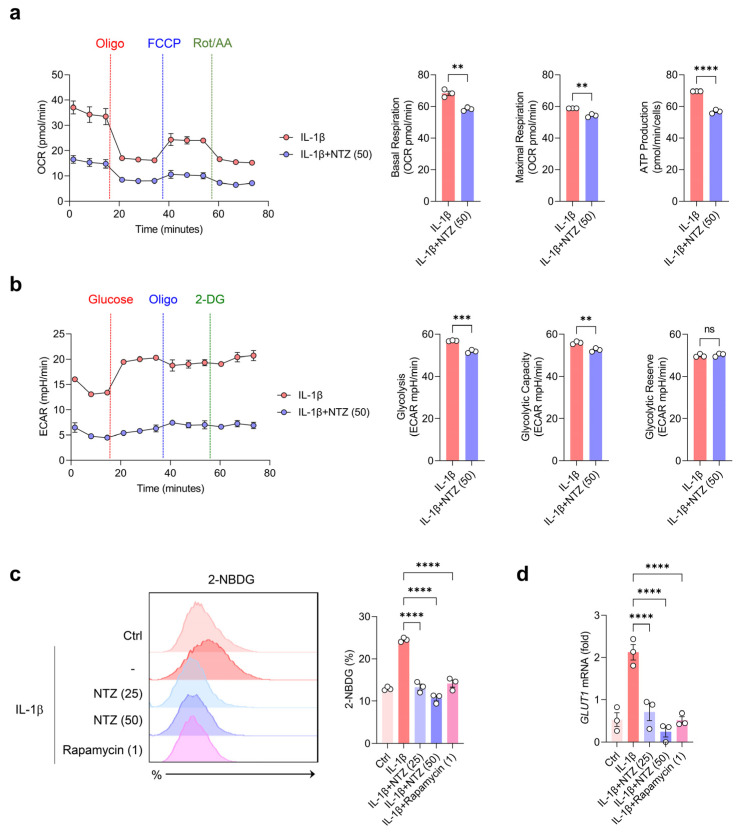
NTZ attenuates IL-1β-induced oxidative phosphorylation (OXPHOS), glycolytic reprogramming, and glucose uptake in OA chondrocytes. Primary chondrocytes derived from OA patients were treated with IL-1β (10 ng/mL) for 24 h, with or without nitazoxanide (NTZ; 25 or 50 μM) or rapamycin (1 μM). (**a**) Mitochondrial respiration was evaluated by measuring the oxygen consumption rate (OCR) using a Seahorse XF analyzer. Quantitative data for basal respiration, maximal respiratory capacity, and ATP production are presented as bar graphs. (**b**) To assess glycolytic activity, the extracellular acidification rate (ECAR) was measured. Glycolysis, glycolytic capacity, and glycolytic reserve were quantified and visualized in bar graphs. (**c**) Glucose uptake was analyzed by flow cytometry following 2-NBDG staining after 2 h of incubation in IL-1β-stimulated chondrocytes, and representative histograms with quantification are shown. (**d**) The expression level of GLUT1 mRNA was determined using qPCR. For (**a**,**b**), statistical significance was assessed using unpaired two-tailed Student’s *t*-tests. For (**c**,**d**), one-way ANOVA followed by Holm–Šídák’s post hoc test was used. Data are presented as mean ± SEM. ** *p* < 0.01, *** *p* < 0.001, and **** *p* < 0.0001 compared to the IL-1β-stimulated group. ns indicates no statistically significant difference.

Metabolic reprogramming of chondrocytes observed under inflammatory conditions is characterized by increased glycolysis and decreased mitochondrial function [19,20]. To investigate the impact of NTZ on mitochondrial function and metabolic activity in OA chondrocytes, oxygen consumption rate (OCR) and extracellular acidification rate (ECAR) were assessed using a Seahorse metabolic flux analyzer. OCR was monitored following the sequential addition of oligomycin (an ATP synthase inhibitor), FCCP (a mitochondrial uncoupler), and rotenone (a complex I inhibitor). Under IL-1β stimulation, chondrocytes exhibited increases in basal respiration, maximal respiratory capacity, ATP generation, proton leak, non-mitochondrial oxygen utilization, spare respiratory capacity, and coupling efficiency. By contrast, all these metrics were consistently decreased in the NTZ treatment group (Figure 2a and Figure S2a). Furthermore, glycolysis, glycolytic capacity, glycolytic reserve, and non-glycolytic acidification, as analyzed using ECAR measurements, remained high in IL-1β but were effectively inhibited by NTZ (Figure 2b and Figure S2b).

To confirm these metabolic inhibitory effects, flow cytometric analysis was conducted using 2-NBDG. IL-1β stimulation substantially increased glucose uptake, and NTZ considerably inhibited IL-1β-induced glucose uptake regardless of concentration (Figure 2c). Rapamycin exhibited a similar inhibitory effect. Subsequent qPCR analysis showed that IL-1β induced the mRNA expression of the glucose transporter, GLUT1, whereas both NTZ and rapamycin substantially inhibited its expression (Figure 2d). NTZ, therefore, restored mitochondrial function and reduced glucose uptake and glycolysis, thereby stabilizing and counteracting the aberrant metabolic programming induced by IL-1β in OA chondrocytes.

### 3.3. NTZ Restores Impaired Mitochondrial Function and Suppresses Oxidative Stress in IL-1β-Induced OA Chondrocytes

**Figure 3:**
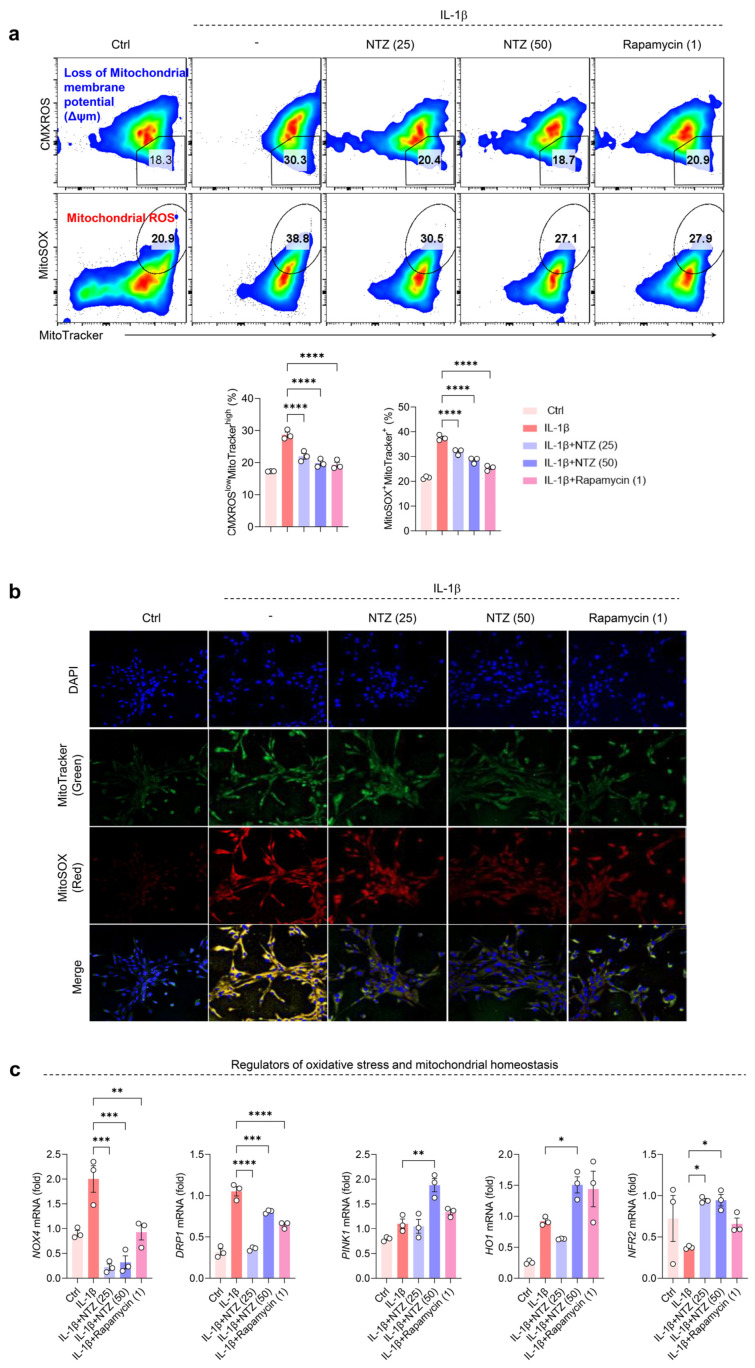
NTZ reverses the accumulation of impaired mitochondria and suppresses mitochondrial oxidative stress production in OA chondrocytes. Primary human chondrocytes derived from OA patients were treated with IL-1β (10 ng/mL) for 24 h, either alone or in combination with NTZ (25 or 50 μM) or rapamycin (1 μM). (**a**) Flow cytometry analysis ((**top panel**): representative plots; (**bottom panel**): quantification) depicts the proportion of cells with dysfunctional mitochondria, identified by low CMXROS and high MitoTracker Green staining, as well as mitochondrial ROS levels (MitoSOX^+^, MitoTracker Green^+^). Δψm and mitochondrial ROS production were assessed using CMXROS and MitoSOX dyes, respectively. (**b**) Confocal fluorescence microscopy images showing mitochondrial content and ROS production in chondrocytes stained with MitoTracker Green and MitoSOX Red. Merged images (yellow) indicate colocalization of mitochondrial content and ROS production. (**c**) Quantitative PCR analysis of genes involved in mitochondrial oxidative stress and redox homeostasis, including *NOX4*, *DRP1*, *PINK1*, *HO*-*1*, and *NRF2*. Statistical significance was determined using the Holm–Šídák post hoc test. Data are presented as mean ± SEM. * *p* < 0.05, ** *p* < 0.01, *** *p* < 0.001, and **** *p* < 0.0001 compared to the IL-1β-stimulated group.

Mitochondrial dysfunction and oxidative stress are closely associated with inflammation-induced cartilage damage, which is one of the main mechanisms underlying OA progression [21]. To evaluate the mitochondrial-protective effects of NTZ, mitochondrial dysfunction and oxidative stress in OA chondrocytes were analyzed. To determine this, MitoTracker Red (CMXROS, Δψm-dependent mitochondrial staining) and MitoTracker Green (Δψm-independent mitochondrial staining) were used together to distinguish between respiring and dysfunctional mitochondria using flow cytometry. IL-1β stimulation increased dysfunctional mitochondria (CMXROS^low^, MitoTracker^high^) in OA chondrocytes, indicating a loss of mitochondrial membrane potential. However, co-treatment with NTZ or rapamycin substantially reduced the loss of membrane potential and restored mitochondrial polarization to near-control levels (Figure 3a). A similar tendency was observed for MitoSOX staining, which reflects oxidative stress levels.

Stimulation with IL-1β markedly increased the proportion of MitoSOX-positive cells, which was substantially attenuated by NTZ and rapamycin treatment (Figure 3a). These results were further confirmed visually using confocal microscopy, which showed a marked decrease in the fluorescence intensity of MitoTracker^+^ and MitoSOX^+^ in the NTZ and rapamycin treatment groups compared to the IL-1β-stimulated group (Figure 3b). This suggests that NTZ inhibited mitochondrial oxidative stress and restored mitochondrial structural integrity.

To further evaluate the molecular mechanism of action of NTZ, we assessed the transcriptional activity of genes involved in mitochondrial function and oxidative stress regulation. Changes in the expression of *NOX4*, *DRP1*, *PINK1*, *HO1*, and *NRF2*, which are involved in mitochondrial function and oxidative stress regulation, were analyzed following NTZ treatment. The expression of *NOX4*, a pro-oxidative stress factor, decreased substantially in OA chondrocytes after NTZ treatment. The expression of *DRP1*, which is associated with mitochondrial fission and dysfunction, was also substantially lower in the NTZ-treated group, suggesting that NTZ inhibits mitochondrial damage. The gene expression of *PINK1*, which is responsible for mitochondrial quality control, as well as *HO1* and *NRF2*, which are key factors in the oxidative stress defense pathway, were substantially increased by NTZ treatment. These results indicate that NTZ may contribute to the inhibition of the pathological deterioration of OA chondrocytes by restoring mitochondrial function and activating antioxidant defense mechanisms (Figure 3c).

### 3.4. NTZ Attenuates mTORC1 Pathway Activation and Concurrently Stimulates AMPK Signaling in Chondrocytes Under IL-1β-Induced Inflammatory Stress

**Figure 4:**
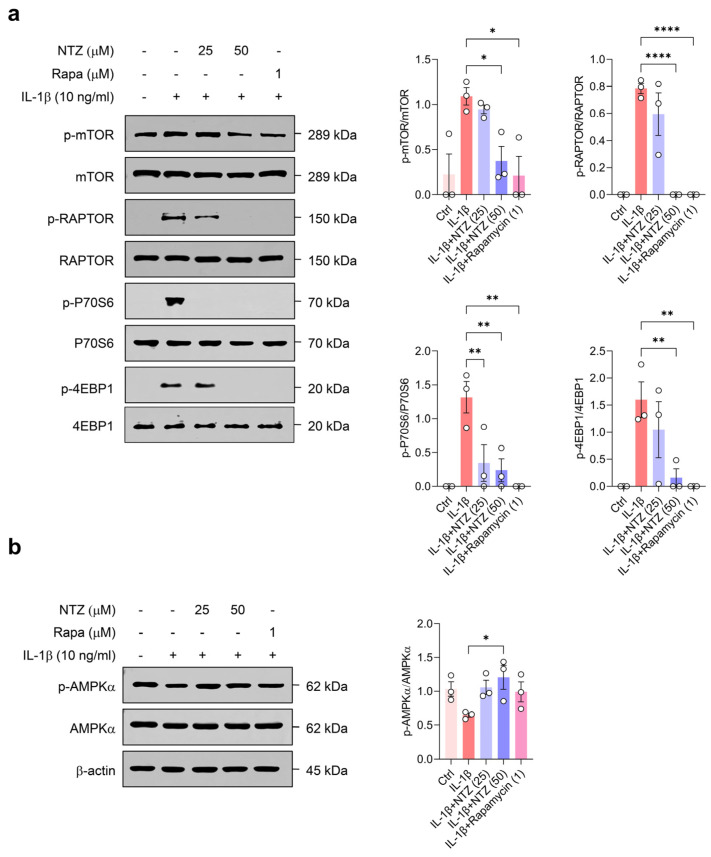
NTZ modulates mTORC1 and AMPK signaling pathways in IL-1β-stimulated OA chondrocytes. Primary chondrocytes derived from OA patients were treated with IL-1β (10 ng/mL) for 24 h, with or without co-treatment with NTZ (25 or 50 μM) or rapamycin (1 μM). Western blot analysis was conducted to show phosphorylation states of (**a**) mTORC1 pathway components (mTOR, RAPTOR, p70S6K, and 4EBP1) and (**b**) AMPKα. The total form and β-actin served as loading controls. Densitometric quantification of protein bands was performed using ImageJ software and is presented as bar graphs. Statistical analysis was conducted using the Holm–Šídák post hoc test. Data are presented as mean ± SEM. * *p* < 0.05, ** *p* < 0.01, and **** *p* < 0.0001 compared to the IL-1β-stimulated group.

Aberrant mTOR signaling has been associated with mitochondrial impairment and cartilage breakdown in osteoarthritis, whereas AMPK activation has been shown to promote mitochondrial biogenesis and enhance oxidative metabolism under inflammatory conditions [22]. Therefore, to elucidate the underlying mechanisms by which NTZ modulates the inflammatory response, mitochondrial function, and oxidative stress in OA chondrocytes, we investigated whether NTZ affects the mTORC1 axis and AMPK activation under IL-1β-induced inflammatory conditions. IL-1β stimulation markedly increased the phosphorylation of mTOR, RAPTOR, p70S6K, and 4EBP1, leading to a strong activation of the mTORC1 pathway (Figure 4a). However, treatment with NTZ (specifically, 50 μM) substantially inhibited the phosphorylation levels of all these mTORC1 components, with similar potency to rapamycin, a well-established mTORC1 inhibitor used as a positive control.

Densitometric analysis indicated that NTZ treatment significantly reduced the phosphorylation status of key mTORC1 pathway proteins, including mTOR, RAPTOR, p70S6K, and 4EBP1, relative to their respective total protein levels (Figure 4a). NTZ treatment substantially increased the phosphorylation of AMPKα (Figure 4b), suggesting that NTZ activated the AMPK pathway to promote energy homeostasis and induce an anti-inflammatory state. These results suggest that NTZ not only mitigates oxidative stress and mitochondrial fragmentation but also attenuates inflammatory signaling in OA chondrocytes through the inhibition of the mTORC1 pathway and the activation of AMPK signaling.

### 3.5. AMPK Activation by NTZ Is an Essential Regulatory Mechanism of mTORC1 Inhibition and Mitochondrial Protective Effects

**Figure 5:**
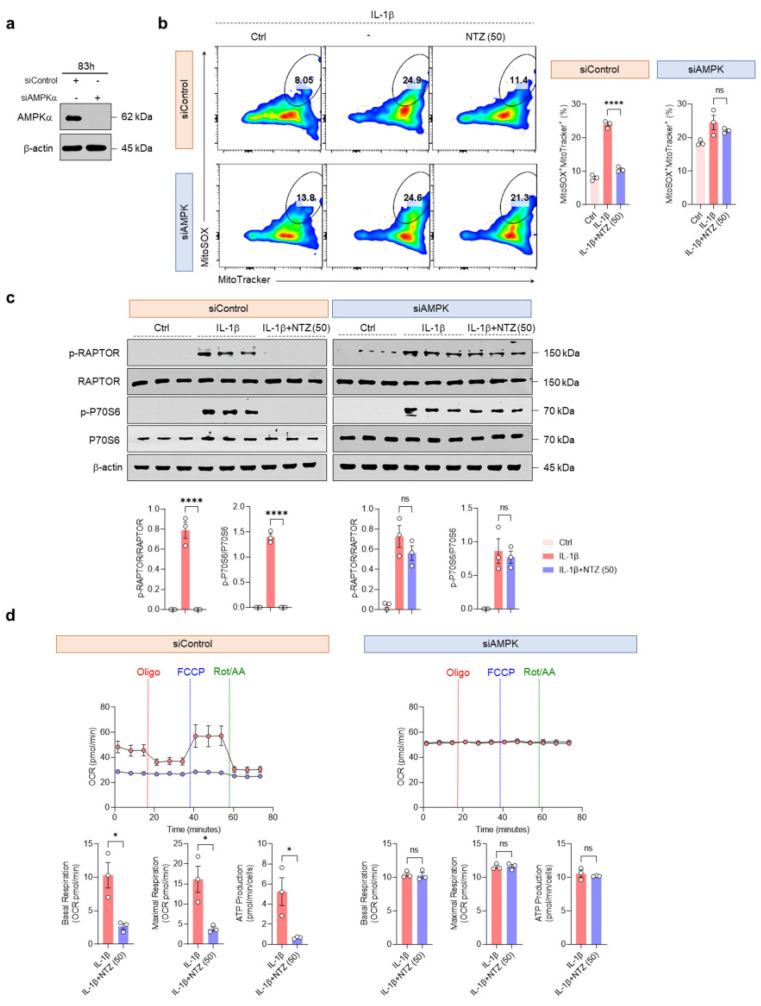
AMPK knockdown negates NTZ-mediated OXPHOS and glycolytic metabolic protection in OA chondrocytes. Primary OA chondrocytes were transfected with either control siRNA or AMPKα-targeting siRNA for 83 h, followed by IL-1β stimulation (10 ng/mL) with or without NTZ (50 μM) for an additional 24 h. (**a**) Western blotting confirmed the efficiency of AMPKα silencing after siRNA transfection. (**b**) Mitochondrial ROS levels were evaluated by flow cytometry after dual staining with MitoTracker Green and MitoSOX Red; representative dot plots (**left**) and quantification graphs (**right**) are shown. (**c**) Protein levels of RAPTOR and P70S6K were assessed by Western blot (**top**), and the band intensities were quantified and plotted (**bottom**). Total protein expression served as the internal loading control. (**d**) Oxygen consumption rate (OCR) profiles were monitored in siControl- and siAMPK-transfected chondrocytes following treatment with oligomycin, FCCP, and rotenone/antimycin A (**top**). Quantitative analysis of mitochondrial respiration parameters, including basal respiration, ATP-linked respiration, and maximal respiratory capacity, is shown in the bar graphs (**bottom**). Statistical significance was determined using the Holm-Šídák post hoc test. Data are presented as mean ± SEM. * *p* < 0.05 and **** *p* < 0.0001 compared to the IL-1β-stimulated group. ns indicates no statistically significant difference.

To assess whether NTZ-mediated mitochondrial protection requires AMPK activity, IL-1β-stimulated OA chondrocytes were transfected with siRNA targeting AMPKα. The effective silencing of AMPK expression was confirmed using western blotting (Figure 5a). Flow cytometry analysis indicated that OA chondrocytes with AMPK knockdown produced mitochondrial ROS more intensely than cells transfected with siControl during IL-1β stimulation. Consistent with Figure 3a, NTZ substantially inhibited mitochondrial ROS production in siControl-transfected cells; however, this inhibitory effect was completely absent in siAMPK-transfected cells (Figure 5b). In line with previous results, Western blotting demonstrated that NTZ substantially inhibited the phosphorylation of RAPTOR and p70S6K in siControl-transfected cells. However, this inhibition was not observed in AMPK-knockdown cells (Figure 5c). This implies that AMPK is an upstream regulator of NTZ-induced mTORC1 inhibition (Figure 5c).

Similar results were observed in terms of metabolic function; in siControl cells, the OCR assay metrics, including basal and maximal respiration, ATP production, non-mitochondrial oxygen consumption, proton leak, spare respiratory capacity, and coupling efficiency, aligned with the findings presented in Figure 2. However, the ability of NTZ to modulate cellular metabolism was entirely lost in AMPK-silenced chondrocytes (Figure 5d and Figure S3a). Furthermore, in ECAR assays, the glycolytic inhibitory effects of NTZ (glycolytic rate, glycolytic capacity, reserve glycolytic capacity, and inhibition of non-glycolytic acidification) were absent in siAMPK cells and remained similar to those of IL-1β-stimulated cells (Figure S3b). These results demonstrate that the mitochondrial protective and metabolic reprogramming inhibitory effects of NTZ in OA chondrocytes were highly dependent on AMPK activation.

## 4. Discussion

OA is more than just a wear-and-tear condition of the articular cartilage; it involves a combination of alterations in cellular metabolism and excessive accumulation of ROS due to decreased mitochondrial function, which promotes disease through inflammatory mediators [8]. Recent studies have identified pathological metabolic reprogramming of chondrocytes and overactivation of mTOR as key mechanisms in the pathogenesis and progression of OA, and novel therapeutic strategies based on the mTOR signaling system and AMPK pathway, a cellular energy sensor, have attracted attention [19,23,24,25]. In this study, we evaluated the therapeutic potential of NTZ, which has previously been used as an antiparasitic agent, for OA and found that NTZ could modulate the AMPK/mTORC1 pathway to maintain energy homeostasis and alleviate mitochondrial ROS accumulation in chondrocytes, thereby ameliorating the inflammatory environment.

Cytokines such as TNF-α, IL-1β, and IL-6 induce joint inflammation and upregulate the production of MMPs (1, 2, 3, 7, 8, 9, and 13) through inflammatory signaling [8]. Under IL-1β stimulation, NTZ significantly suppressed the expression of key pro-inflammatory mediators, including TNF-α, IL-6, and IL-1β, in human chondrocytes and substantially reduced the expression of MMP1, MMP3, and MMP13 proteins, indicating a protective effect that extends beyond simple cytokine inhibition and prevents cartilage degradation through inhibition of matrix-degrading enzymes. NTZ has demonstrated anti-inflammatory potential in multiple inflammatory disease models by downregulating NF-κB signaling and reducing the expression of key cytokines such as IL-1β and IL-6. It has also been shown to suppress osteoclast differentiation [15,16]. The present data indicate that NTZ retains its inflammation-suppressing properties in the context of OA chondrocytes.

Cartilage energy metabolism is primarily sustained by glucose and oxygen as key substrates. Glycolysis serves as the dominant pathway for ATP production, accounting for over 75% of energy generation, while the remaining portion is derived from mitochondrial oxidative phosphorylation (OXPHOS) [20]. Glucose transport in chondrocytes is mainly facilitated by GLUT1, and elevated glucose levels have been implicated in promoting cartilage catabolic activity [26]. In IL-1β-stimulated OA chondrocytes, this bioenergetic profile is altered, with increased levels of glycolysis alongside increased basal and maximal respiration [26]. Thus, NTZ normalized mitochondrial respiratory capacity by restoring the OCR and reducing the ECAR, and reversed the reprogramming of energy metabolism in the inflammatory environment of OA through glycolysis and inhibition of GLUT1 expression. These effects of NTZ are consistent with a previous report that NTZ reduced intracellular glucose metabolism and ATP production in a psoriatic inflammatory model [15].

As OA progresses, mitochondrial dysfunction becomes evident through structural alterations, disrupted dynamics, and compromised genomic integrity. These changes contribute to impaired respiratory capacity, elevated production of reactive oxygen species (ROS), and increased oxidative stress in chondrocytes [20]. In OA patient-derived chondrocytes, disturbances in mitochondrial membrane potential have been reported, and cytokine-induced ROS further promote the persistence of dysfunctional mitochondria [20,27]. Such mitochondrial disturbances drive cartilage degeneration in OA, and targeting ROS with antioxidant strategies may help preserve cartilage integrity and suppress inflammation in chondrocytes [19,28,29]. Our results demonstrate that NTZ has mitochondrial protective effects. Using the MitoTracker, CMXROS, and MitoSOX assays, NTZ was found to restore dysfunctional mitochondria and inhibit ROS production, suggesting that NTZ fundamentally reduces mitochondrial damage and oxidative stress in chondrocytes. The inhibition of NOX4 and DRP1, which are major sources of ROS, was accompanied by increased expression of PINK1, NRF2, and HO1 [30], suggesting that NTZ has a multifaceted protective mechanism that activates mitochondrial division inhibition and antioxidant signaling. Metabolic changes are attributed to the dysregulation of mitochondria and ROS production, which are responsible for energy production, as well as the dysregulation of energy-sensing signaling pathways such as mTOR and AMPK [19,20]. In particular, the activation of mTORC1 signaling promotes protein overproduction in chondrocytes, inhibits autophagy, and induces various stress responses, including growth factor stimulation, DNA damage, hypoxia, and abnormal ATP levels [19]. In the present study, NTZ restored the balance of the energy-sensing system in chondrocytes by inhibiting the phosphorylation of RAPTOR, p70S6K, and 4EBP1, which are the component proteins of mTOR and mTORC1, while inducing AMPK activity. Inhibiting the expression of mTOR in chondrocytes increases the expression of AMPK [31], and restoring AMPK activity reduces inflammation and prevents the progression of OA [8]. Recent studies have emphasized the role of AMPK-mTOR signaling in maintaining joint homeostasis and in the pathogenesis of OA. Dysregulation of this axis has been reported to impair autophagy, promote mitochondrial dysfunction, and enhance cartilage degradation metabolism [32,33,34]. Notably, activation of AMPK via agents such as metformin and β-hydroxybutyrate inhibited cartilage damage in OA by restoring mitochondrial function and suppressing joint inflammation [33,34,35]. Thus, consistent with these findings, our results strongly support the repurposing of NTZ as a modulator targeting the AMPK-mTOR pathway in the inflammatory context of OA.

Furthermore, the AMPK agonist, AICAR, inhibits the catabolic response of chondrocytes to inflammatory cytokines, whereas AMPK depletion increases the inflammation-induced chondrolytic response [8,19,36]. The impairment of AMPK activity exacerbates mitochondrial dysfunction and leads to excessive ROS production [19].

The results suggest that NTZ can positively regulate the AMPK/mTORC1 axis, thereby inhibiting mTORC1 signaling, suppressing mitochondrial ROS production, and reversing inflammation-altered energy metabolism. AMPK knockdown experiments using siRNA clearly demonstrated that the effect of NTZ was dependent on AMPK activity, suggesting that NTZ could be utilized as a novel therapeutic strategy based on metabolic regulation in OA chondrocytes.

Although NTZ was developed as an antiparasitic drug, its antiviral, antioxidant, anti-inflammatory, and anti-cancer effects are being evaluated for repurposing in a variety of diseases. NTZ has recently gained attention for its therapeutic potential in conditions such as rheumatoid arthritis, COVID-19, Alzheimer’s disease, and Parkinson’s disease [16,37,38,39]. As an FDA-approved drug, NTZ exhibits favorable bioavailability and a well-documented safety profile, supporting its exploration as a candidate for repurposing in osteoarthritis treatment; therefore, the efficacy of NTZ in OA chondrocytes presented in this study may provide a basis for its repurposing as a novel disease-modifying osteoarthritis drug (DMOAD). In addition to chondrocytes, NTZ also showed anti-inflammatory effects in OA patient-derived fibroblast-like synoviocytes (FLS) (Figure S4), suggesting the potential for NTZ to exert broad protective effects across multiple joint-resident cell types.

This study focused on the short-term effects of NTZ in OA patient-derived chondrocytes. As AMPK activation is closely associated with autophagy, it is necessary to further explore whether autophagy is regulated in vitro and in vivo to determine the long-term effects of NTZ. Future studies may further clarify the chondroprotective mechanism of NTZ by analyzing autophagic flux and related markers.

## 5. Conclusions

Our findings indicate that NTZ plays a protective role against multiple aspects of OA pathology through AMPK-dependent modulation of metabolic abnormalities and mitochondrial dysfunction induced by inflammatory stimuli in chondrocytes derived from patients with OA. These findings suggest that NTZ is a promising DMOAD candidate that can target the pathophysiology of OA by restoring metabolic homeostasis, improving mitochondrial function, and modulating the AMPK/mTOR metabolic signaling pathway beyond its simple anti-inflammatory activity. These results support the drug-repurposing potential of NTZ, and its therapeutic potential should be evaluated in future preclinical and clinical studies.

## Data Availability

The data supporting the findings of this study are available from the corresponding author upon reasonable request.

## Supplementary Materials

The following supporting information can be downloaded at: https://www.mdpi.com/article/10.3390/antiox14050512/s1. Figure S1: Chemical structure of nitazoxanide (NTZ) and cell viability assay in OA chondrocytes. Figure S2: Detailed analysis of oxidative phosphorylation (OXPHOS) and glycolysis in interleukin (IL)-1β-stimulated OA chondrocytes treated with NTZ. Figure S3: AMPK knockdown negates NTZ-mediated metabolic regulation in OA chondrocytes. Figure S4: NTZ attenuates pro-inflammatory and catabolic gene expression in IL-1β-stimulated OA fibroblast-like synoviocytes (FLS). Table S1: Forward and reverse primer sequences for quantitative PCR analysis.

## Abbreviations

The following abbreviations are used in this manuscript:

OA	osteoarthritis
NTZ	nitazoxanide
MMPs	matrix metalloproteinases
TNF-α	tumor necrosis factor-α
IL	interleukin
ROS	reactive oxygen species
mTOR	mammalian target of rapamycin
mTORC1	mTOR complex 1
FDA	Food and Drug Administration
SF DMEM	serum-free Dulbecco’s modified Eagle medium
FBS	fetal bovine serum
MTT	3-(4,5-Dimethylthiazol-2-yl)-2,5-diphenyltetrazolium bromide
OCR	oxygen consumption rate
ECAR	extracellular acidification rate
PBS	phosphate-buffered saline
DPBS	Dulbecco’s PBS
DAPI	diamidino-2-phenylindole
qPCR	quantitative real-time PCR
OXPHOS	oxidative phosphorylation
DMOAD	disease-modifying osteoarthritis drug
FLS	fibroblast-like synoviocytes
