# Supplementary material for: Nitazoxanide Modulates Mitochondrial Function and Inflammatory Metabolism in Chondrocytes from Patients with Osteoarthritis via AMPK/mTORC1 Signaling
Source: Antioxidants (Basel). 2025 Apr 24;14(5):512. doi: 10.3390/antiox14050512 (PMC12108455; doi:10.3390/antiox14050512)
Supplement: Supplementary file 1 [file antioxidants-14-00512-s001.zip › antioxidants-3596631-supplementary.pdf]

Supplementary Information for

Nitazoxanide modulates mitochondrial function and inflam-matory metabolism in chondrocytes from patients with osteoarthritis via AMPK/mTORC1 signaling

Ha Eun Kim, Jong Yeong Lee, Ga-Yeon Son, Jun-Young Park, Ki Bum Kim, Young Jae Moo n, Jin Kyeong Choi

Corresponding author: Young Jae Moon and Jin Kyeong Choi

E-mail: yjmoonos@jbnu.ac.kr; jkchoi@jbnu.ac.kr

Files includes:

Figures S1 – S4

Table S1

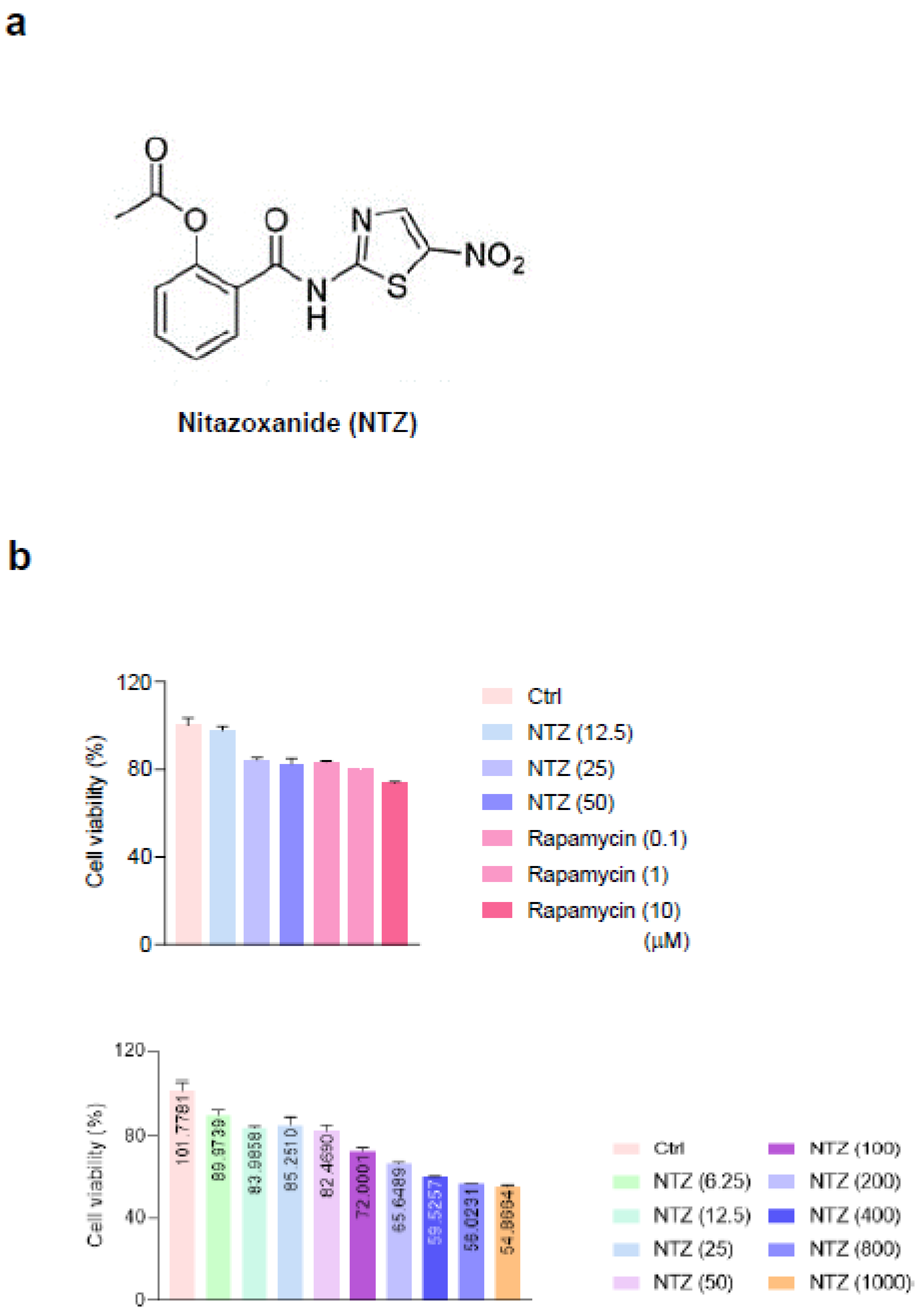

Figure S1. Chemical structure of Nitazoxanide (NTZ) and cell viability assay in osteoarthritis (OA) chondrocytes. (a) Chemical structure of NTZ. (b) MTT assay assessing cell viability of OA chondrocytes treated with NTZ (6.25-1000 μM) or rapamycin (0.1, 1, and 10 μM) for 24 h. Concentrations maintaining cell viability above 80% were selected for subsequent experiments.

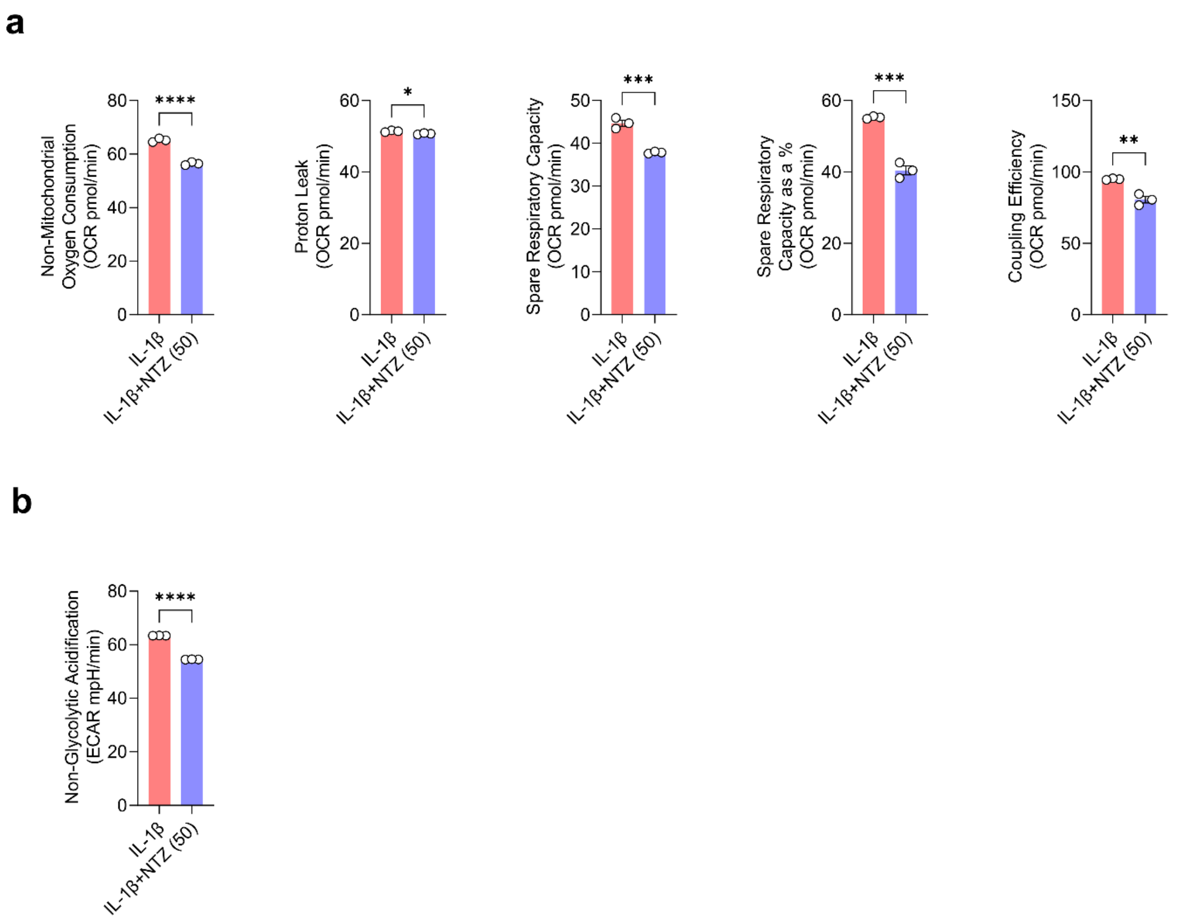

Figure S2. Detailed analysis of oxidative phosphorylation (OXPHOS) and glycolysis in interleukin (IL)-1β-stimulated OA chondrocytes treated with NTZ. Primary human chondrocytes isolated from patients with OA were stimulated with IL-1β (10 ng/mL) for 24 h in the presence or absence of NTZ (50 μM). (a) Real-time changes in oxygen consumption rate (OCR) of chondrocytes in response to oligomycin, FCCP, and Rot/AA. Bar charts showing nonmitochondrial oxygen consumption, proton leak, spare respiratory capacity, spare respiratory capacity, and coupling efficiency. (b) Real-time changes in extracellular acidification rate (ECAR) of OA chondrocytes in response to glucose, oligomycin, and 2-DG. Bar chart showing non-glycolytic acidification. Statistical analysis was conducted using the Holm-Šídák post-hoc test. Data are presented as mean ± standard error of mean (SEM). $*P < 0.05$, $***P < 0.001$, and $****P < 0.0001$ compared to the IL-1β-stimulated group

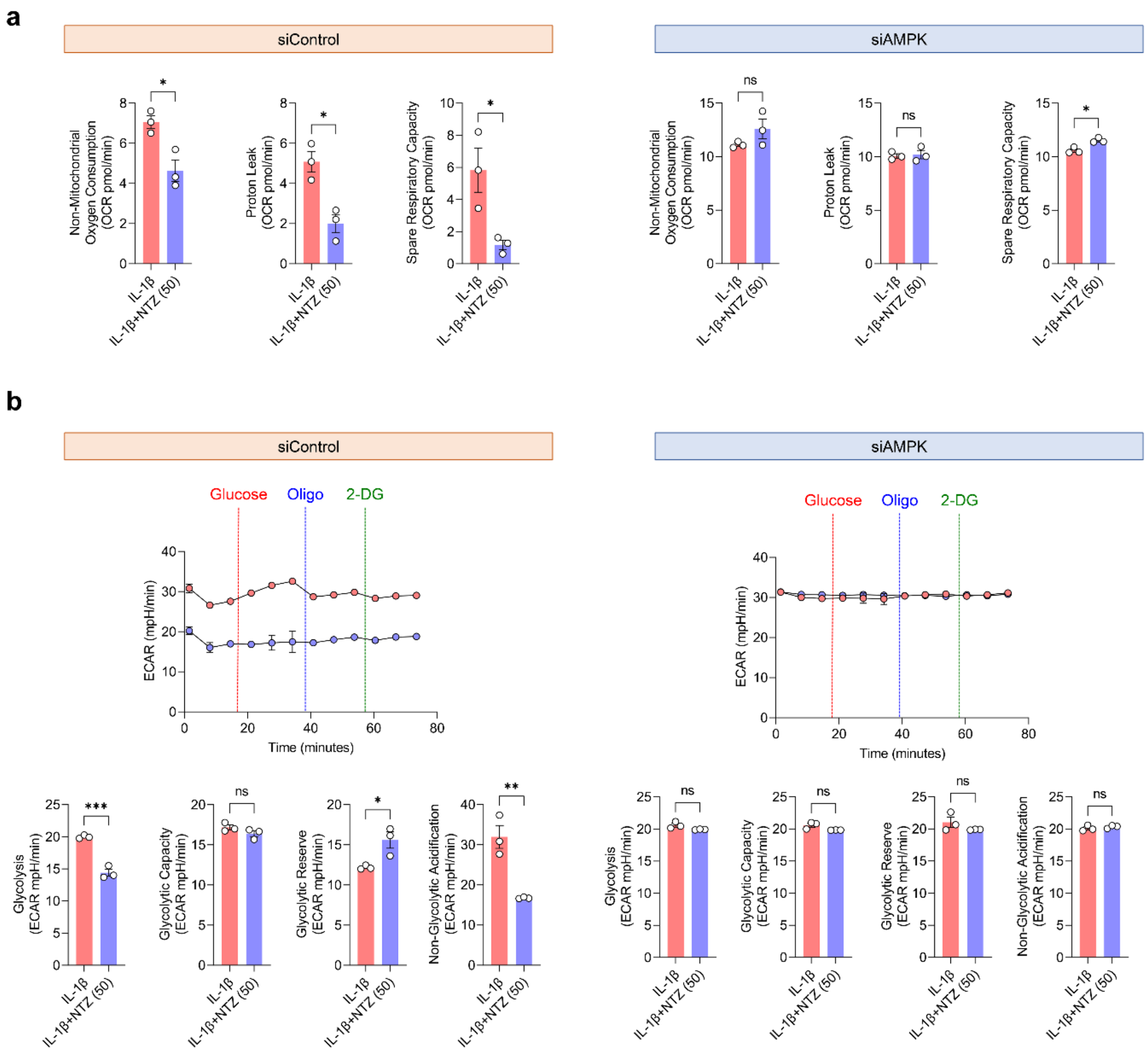

Figure S3. AMPK knockdown negates NTZ-mediated metabolic regulation in OA chondrocytes. Primary human chondrocytes isolated from patients with OA were transfected with control or AMPK siRNA for 83 h and then stimulated with IL-1β (10 ng/mL) in the presence or absence of NTZ (50 µM) for 24 h. (a) Real-time changes in OCR of chondrocytes in response to oligomycin, FCCP, and Rot/AA. Bar charts show nonmitochondrial oxygen consumption, proton leak, and spare respiratory capacity. (b) Real-time changes in ECAR of OA chondrocytes in response to glucose, oligomycin, and 2-DG. Bar charts show glycolysis, glycolytic capacity, glycolytic reserve, and non-glycolytic acidification. Statistical analysis was conducted using the Holm-Šídák post-hoc test. Data are presented as mean ± SEM. $^{*}P < 0.05$, $^{**}P < 0.01$, and $^{***}P < 0.001$ compared to the IL-1β-stimulated group.

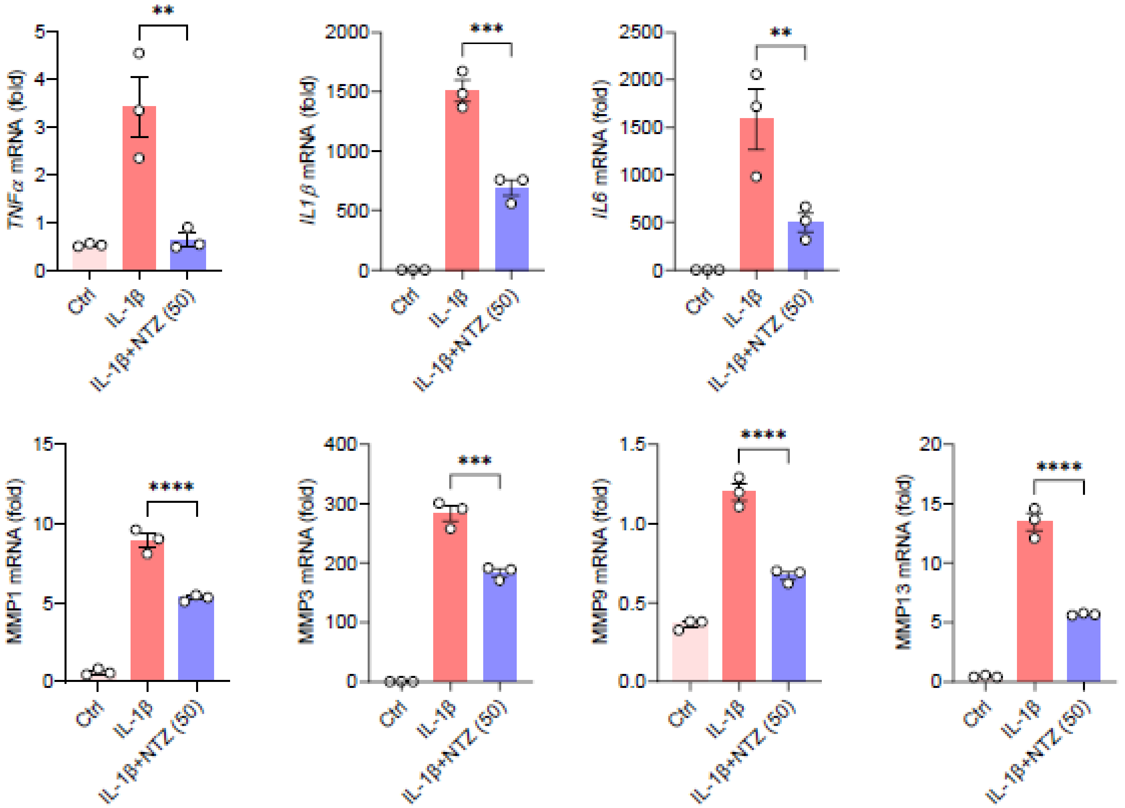

Figure S4. NTZ attenuates pro-inflammatory and catabolic gene expression in IL-1β-stimulated OA fibroblast-like synoviocytes (FLS). Primary FLS isolated from OA patients were stimulated with IL-1β (10 ng/mL) in the presence or absence of NTZ (50 µM) for 24 hours. mRNA expression levels of pro-inflammatory cytokines (*TNF-α, IL-1β,* and *IL-6*) and matrix metalloproteinases (*MMP1, MMP3, MMP9*, and *MMP13*) were quantified by qPCR. NTZ significantly reduced the expression of all tested genes compared to IL-1β stimulation alone, indicating its broad anti-inflammatory and anti-catabolic effects in joint-resident FLS. Statistical analysis was conducted using the Holm-Šídák post-hoc test. Data are presented as mean ± SEM. **$P < 0.01$, ***$P < 0.001$, ****$P < 0.0001$ compared to the IL-1β-stimulated group.

Supplementary Table S1. Forward and reverse primer sequences for quantitative PCR analysis

Human

Gene	Accession Number	Forward primer (5'>3')	Reverse primer (5'>3')
TNFα	NM_000594.3	GAGCTGAGAGATAACCA GCTGGTG	CAGATAGATGGGCTCATA CCAGGG
IL1β	NM_000576.3	GCTGATGGCCCTAAACAG ATGAA	TGAAGCCCTTGCTGTAGT GGTG
IL6	NM_000600.5	CCCCTGACCCAACCACA AAT	CATTTGCCGAAGAGCCCT CA
MMP1	NM_002421.4	ATGAAGCAGCCCAGATG TGGAG	TGGTCCACATCTGCTCTT GGCA
MMP3	NM_002422.4	TTCCTTGGATTGGAGGTG AC	TGCCAGGAAAGGTTCTG AAG
MMP9	NM_004994.3	GCCACTACTGTGCCTTTG AGTC	CCCTCAGAGAATCGCCA GTACT
MMP13	NM_002427.4	CTATGGTCCAGGAGATGA AG	AGAGTCTTGCCTGTATCC TC
GLUT1	NM_006516.4	AACTCTTCAGCCAGGGT CCAC	CACAGTGAAGATGATGA AGAC
DRP1	NM_005690.4	GATGCC ATAGTTGAAGTG GTGAC	CCACAAGCATCAGCAAA GTCTGG
NFR2	NM_006164.5	CACATCCAGTCAGAAAC CAGTGG	GGAATGTCTGCGCCAAA AGCTG
MFN2	NM_014874.5	ATTGCAGAGGCGGTTCG ACTCA	TTCAGTCGGTCTTGCCGC TCTT
NOX4	NM_016931.4	TTGGGGCTAGGATTGTGT CTA	GAGTGTTCGGCACATGG GTA
PINK1	NM_032409.2	CAAGAGAGGTCCCAAGC AAC	GGCAGCACATCAGGGTA GTC
HO1	NM_002133.3	CCAGGCAGAGAATGCTG AGTTC	AAGACTGGGCTCTCCTTG TTGC
β-actin	NM_001101.5	AGAGCTACGAGCTGCCT GAC	AGCACTGTGTTGGCGTAC AG
